# Distinct biogeographical patterns in snail gastrointestinal tract bacterial communities compared with sediment and water

**DOI:** 10.1002/mbo3.1413

**Published:** 2024-06-02

**Authors:** Daniel P. R. Herlemann, Helen Tammert, Carmen Kivistik, Kairi Käiro, Veljo Kisand

**Affiliations:** ^1^ Centre for Limnology, Institute of Agricultural and Environmental Sciences Estonian University of Life Sciences Tartu County Estonia; ^2^ Department of Biological Oceanography Leibniz Institute for Baltic Sea Research Warnemünde (IOW) Rostock Germany; ^3^ Institute of Technology University of Tartu Tartu Estonia

**Keywords:** habitat island, holobiont, host‐associated, symbiont

## Abstract

The factors that influence the distribution of bacterial community composition are not well understood. The role of geographical patterns, which suggest limited dispersal, is still a topic of debate. Bacteria associated with hosts face unique dispersal challenges as they often rely on their hosts, which provide specific environments for their symbionts. In this study, we examined the effect of biogeographic distances on the bacterial diversity and composition of bacterial communities in the gastrointestinal tract of *Ampullaceana balthica*. We compared the effects on the host‐associated bacterial community to those on bacterial communities in water and sediment. This comparison was made using 16S ribosomal RNA gene sequencing. We found that the bacterial communities we sampled in Estonia, Denmark, and Northern Germany varied between water, sediment, and the gastrointestinal tract. They also varied between countries within each substrate. This indicates that the type of substrate is a dominant factor in determining bacterial community composition. We separately analyzed the turnover rates of water, sediment, and gastrointestinal bacterial communities over increasing geographic distances. We observed that the turnover rate was lower for gastrointestinal bacterial communities compared to water bacterial communities. This implies that the composition of gastrointestinal bacteria remains relatively stable over distances, while water bacterial communities exhibit greater variability. However, the gastrointestinal tract had the lowest percentage of country‐specific amplicon sequence variants, suggesting bacterial colonization from local bacterial communities. Since the overlap between the water and gastrointestinal tract was highest, it appears that the gastrointestinal bacterial community is colonized by the water bacterial community. Our study confirmed that biogeographical patterns in host‐associated communities differ from those in water and sediment bacterial communities. These host‐associated communities consist of numerous facultative symbionts derived from the water bacterial community.

## Graphical abstract

This study investigates the biogeographical patterns of bacterial communities in the gastrointestinal tract of *Ampullaceana balthica* snails compared with those in sediment and water. The findings indicate that substrate type significantly influences bacterial community composition, with distinct patterns observed in snail gastrointestinal tract communities. The research highlights the role of local water bacterial communities in colonizing the gastrointestinal tract, contributing to our understanding of host‐associated bacterial biogeography.

## 1 INTRODUCTION

Bacterial symbiosis allows hosts to colonize otherwise unfavorable habitats and increases their metabolic capacities (Bordenstein & Theis, 2015). These intimate host–microbe relationships have led to the concept of “holobionts” where the symbiosis is understood as a single ecological unit (Margulis & Fester, 1991). The gut microbiota is a multilayered structure, composed of both a host‐adapted (“permanent”) microbiota under host genetic and immune control and a flexible pool of microbes modulated by the environment (“transient microbiome”) (Macke et al., 2017). Permanently present bacteria are often responsible for influencing the host development, aiding digestion or supplementing the nutrient pool, supporting the immune system, and influencing both physiology and metabolism (Nayak, 2010; Nicolai et al., 2015; Pinheiro et al., 2015; Sommer & Bäckhed, 2013). Transient bacteria can also have a strong influence on the host, especially during stressful situations (Kivistik et al., 2022b). The symbionts are acquired from a combination of environmental (horizontal transfer) and parent‐to‐offspring (vertical transfer) colonization of the host microbiome (Bright & Bulgheresi, 2010), depending on the host species. Vertical transmission has a direct influence on the gastrointestinal bacterial community and results in relatively structured populations (Nyholm & McFall‐Ngai, 2004). These type of populations are typically found in eusocial animals, such as termites. Despite a long history of research on host‐associated microbial communities, biogeographical patterns of host‐associated microbiomes within one climatic zone are not well investigated and understood (Härer & Rennison, 2023; Schellenberg & Clarke, 2020), especially for organisms with relatively unstructured gastrointestinal microbiomes.

The principles responsible for microbial distributions are widely debated (Hanson et al., 2012). The Baas Becking hypothesis that “everything is everywhere, but the environment selects” has predominated among microbiologists (Baas Becking, 1931; O'Malley, 2007). Under this assumption, microorganisms disperse freely, unhindered by biogeographical boundaries, settling and thriving after encountering favorable environments (De Wit & Bouvier, 2006). However, modern methods in molecular biology have provided microbiologists with a more detailed picture of microbial diversity, indicating that microorganisms are not as mobile or ubiquitous as previously believed and that many display biogeographical patterns (Eiler et al., 2011; Lowe et al., 2012; Zwirglmaier et al., 2008). Biogeographical patterns over various taxonomic and spatial scales have been shown for water bacterial communities (García‐Martínez & Rodríguez‐Valera, 2000; Hellweger et al., 2014; Riemann & Middelboe, 2002; Schwalbach & Fuhrman, 2005). In these studies, dispersal barriers and past climatic conditions lead to genetic divergence and subsequent variation in biogeographical distributions (Cox et al., 2016). The existence of such provinces has been addressed with a biogeographical pattern emerging among various bacteria (Casteleyn et al., 2010; Lowe et al., 2012; Martiny et al., 2006; Pommier et al., 2007). Highly diverse and specific host‐associated microbial communities are important reservoirs for biodiversity (Taylor et al., 2005). Aquatic host organisms offer distinct environmental conditions for microbial colonization compared to the surrounding environment (Suzzi et al., 2023). They function as habitat islands (MacArthur & Wilson, 2001) and provide unique ecological niches. This allows the allopatric speciation of gastrointestinal bacterial communities living in physically separated hosts resulting in distinct gastrointestinal bacterial communities and increased distance‐decay patterns (Papke & Ward, 2004; Taylor et al., 2005). However, allopatric speciation also depends on environmental filtering, for example, through diet and physiochemical factors that shape host‐associated bacterial communities (Kivistik et al., 2022a, 2022b; Linnenbrink et al., 2013; Terraneo et al., 2019), especially in organisms without vertically transferred gastrointestinal bacterial communities. Hence the host‐associated bacterial community composition is typically a combination of host characteristics and environmental factors (Costello et al., 2012; Krotman et al., 2020; Ley et al., 2008; Sylvain et al., 2019). Alternatively, geographically isolated populations occur and speciate separately, resulting in endemic populations (Taylor et al., 2005).

Snail gastrointestinal bacterial communities harbor distinct bacterial assemblages from the environment and represent an easily accessible model organism for aquatic gastrointestinal bacterial communities (Kivistik et al., 2020, 2022a, 2022b). The common pond snail *Ampullaceana balthica* (Linnaeus 1758) is a Palearctic species widely distributed in Eurasian biogeographic regions, spreading from Iceland (Mandahl‐Barth, 1938) and Norway (Økland, 1990) in the north, northern Africa in the south (Brown, 2002; Van Damme, 1984), Ireland and Spain in the west, and up to southern Siberia in the east. Aksenova et al. (Aksenova et al., 2018) placed the former *Radix balthica* in the genus *Ampullaceana* Servain, 1881 based on molecular genetics. This freshwater species prefers hard‐bottomed low‐altitude running and standing freshwater, such as lakes, ponds, drainage ditches, and lentic zones of rivers, which are rich in nutrients and submerged vegetation (Glöer & Diercking, 2010). *A. balthica* feeds on green algae, detritus, diatoms, Cyanobacteriota, and protists (Knecht & Walter, 1977; Reavell, 1980). Its gastrointestinal bacterial community composition is relatively variable with few potentially specific bacteria suggesting mostly horizontal transmission of the gastrointestinal bacterial community (Kivistik et al., 2022a). Therefore, this model system differs from previously studied aquatic model systems with a strong host‐control, like, sponges and corals (Baldassarre et al., 2022, 2023; Lesser et al., 2016; Webster et al., 2013).

In this study, we investigated the diversity and biogeography of the host‐associated bacterial community composition of *A. balthica* sampled in Estonia, Denmark, and Northern Germany and compared it with the nearby water and sediment bacterial communities. We hypothesized that the *A. balthica* gastrointestinal tract acts as a habitat island, that is, a niche where specific bacteria occur due to limited access. This is indicated in different bacterial community compositions compared with sediment and water and a lower distance‐decay relationship of host‐associated bacteria compared with sediment and water habitats.

## 2 MATERIALS AND METHODS

### 2.1 Sampling

**Figure 1:**
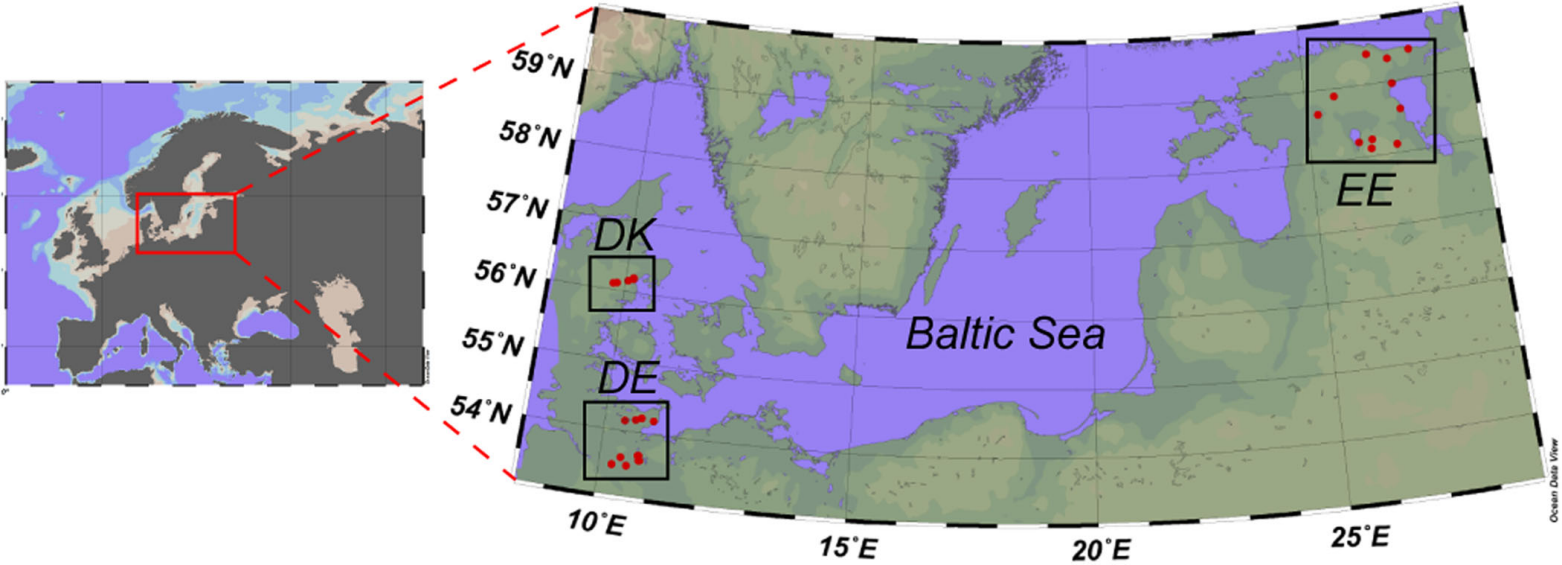
Map of the sampling sites in Denmark (DK), Germany (DE), and Estonia (EE).

Three sediment and water samples and four snails for gastrointestinal microbiome samples were collected from each sampling site in Denmark (DK), Estonia (EE), and Germany (DE) (Table 1 and Figure 1). The sampling stations in each country were closer to each other than to any sampling station in the other countries. Sediment was collected in sterile 2 mL tubes by carefully scraping the top 1 cm of the sediment (macrophytes or litter were removed if present). Water was collected using 100 mL syringes and pumped through Sterivex GP 0.2 µm filters on site. Sediment and water filters were stored cooled (4°C) for transport (2–3 h) and were kept frozen at −20°C until DNA extraction. Snails were placed into tap water for 24 h to reduce the number of food‐derived bacteria in the microbiome and frozen at −20°C until dissection. *A. balthica* samples included average snails of age, size, and health status. Before dissecting the gastrointestinal tract, the shell was treated with 70% ethanol to avoid external contamination. The dissection process was performed under sterile conditions on Petri dishes. The snail's soft body was carefully removed from the shell with tweezers, and the gastrointestinal tract was isolated from the rest of the body.

**Table 1:** Location, country, sampling time, and type of sampling sites.

Name	Country	Date	Latitude	Longitude	Type
Brendstrup	Denmark	October 2021	56.1871	10.1652	Flowing
Koldkar	Denmark	October 2021	56.2035	10.1600	Standing
Årslev	Denmark	October 2021	56.1596	10.0557	Flowing
Lillesø	Denmark	December 2021	56.0941	9.7453	Standing
Ravnsø	Denmark	December 2021	56.1051	9.8204	Standing
Võrtsjärv	Estonia	September 2021	58.2120	26.1094	Standing
Verevi	Estonia	September 2021	58.2288	26.4050	Standing
Esna	Estonia	September 2021	58.8927	25.6708	Flowing
Ahja	Estonia	September 2021	58.1238	26.9579	Flowing
Pühajõgi	Estonia	September 2021	59.4032	27.5363	Flowing
Karjamõisa	Estonia	September 2021	58.1065	26.3708	Standing
Avijõgi	Estonia	September 2021	58.9661	27.0294	Flowing
Purtse	Estonia	September 2021	59.3194	27.0099	Flowing
Kunda	Estonia	September 2021	59.4185	26.5392	Flowing
Pärnu	Estonia	September 2021	58.6655	25.2554	Flowing
Randkanal	Germany	September 2021	54.1885	10.9859	Flowing
Kellersee	Germany	September 2021	54.1834	10.6171	Standing
Mönchnevers	Germany	September 2021	54.2210	10.7389	Standing
Lüttau	Germany	September 2021	53.6028	10.7083	Standing
Schwentine	Germany	September 2021	54.1534	10.4052	Flowing
Schulenbrooksbek	Germany	September 2021	53.4843	10.2642	Flowing
Müssen	Germany	September 2021	53.4899	10.5523	Standing
Ratzeburg	Germany	September 2021	53.6821	10.7537	Standing
Trittau	Germany	September 2021	53.6074	10.4167	Flowing

### 2.2 DNA extraction

Different DNA extraction protocols were used to optimize the yield of genomic DNA from each sample. For DNA extraction, the frozen snails were melted for 10 min at room temperature, and after dissection, DNA extraction was performed based on a modified protocol from Lueders et al. (2004) and Weinbauer et al. (2002) as described in Kivistik et al. (2020). In brief, gastrointestinal tracts were incubated at 65°C for 1 h and bead‐beaten for 3 min at 2000 rpm. From the supernatant, DNA was extracted using a mixture of phenol:chloroform:isoamyl alcohol (25:24:1). The DNA extract was incubated with RNase (100 mg/mL) (Qiagen), washed with isopropanol and with 95% ethanol. The pellet was resuspended in 50 μL AE buffer (Qiagen). For water bacterial community analysis, complete filters were removed from the Sterivex capsules and cut into six pieces under sterile conditions. DNA extraction was performed by using the DNeasy PowerWater Kit (Qiagen) following the producer's instructions. Sediment (250 mg) was transferred into bead‐beating columns provided in the PowerSoil Pro Kit (Qiagen) and DNA was extracted according to the manufacturer's protocol.

### 2.3 Bacterial community analysis

For bacterial community analysis, the DNA was polymerase chain reaction (PCR) amplified using V3–V4 primer amplifying the 16S ribosomal RNA (rRNA) gene according to the protocol of Herlemann et al. (2011) and Kivistik et al. (2020). The amplicons were purified using PCR Kleen (Bio‐Rad), tags were added, and sent to FIMM, University of Helsinki, Finland for Illumina (MiSeq) sequencing using PE250 chemistry.

### 2.4 Phylogenetic analysis of *A. balthica*

For the phylogenetic assignment of the snails, the internal transcribed spacer 2 (ITS2) was analyzed. PCR on genomic DNA from snails was performed according to a protocol from (Vinarski et al., 2011) using the primers LT1 (Bargues et al., 2001) and ITS2‐Rixo (Almeyda‐Artigas et al., 2000). The temperature profile used was 94°C 4 min, 35 × (94°C 30 s, 56°C 30 s, 72°C 1 min), 72°C 7 min, 8°C store. The amplicons were purified using PCR Kleen (Bio‐Rad) and Sanger sequenced by the sequencing facility of the University of Tartu, Estonia.

### 2.5 Bioinformatic processing of the sequences

The raw sequence data from Illumina MiSeq sequencing were processed with the QIIME2 microbiome analysis package (Bolyen et al., 2019) using default settings. Quality filtering and *Chimaera* identification were performed using the DADA2 plugin for QIIME2 (Callahan et al., 2016) with SILVA 138.1 as the database. Sequences assigned to chloroplasts, mitochondria, eukaryotes, and Archaea were excluded because the primer set employed in the analysis had very limited coverage of these groups. Samples with insufficient amplicon products for sequencing, lower quality sequencing results, and samples with less than 1000 reads after removal of chloroplasts, mitochondria, eukaryotes, and Archaea were excluded from the analysis. This resulted in a total of 3,301,247 reads assigned to 4787 amplicon sequencing variants (ASVs) for 63 sediment samples, 62 water samples, and 88 snail samples. The raw reads were deposited at the NCBI in bioproject PRJNA1028832 under the accession numbers SAMN38726042–SAMN38726255.

Water and gastrointestinal samples from Ratzeburg and Lütau did not yield sufficient high‐quality reads and were therefore excluded from the analysis. The sequence names of SILVA 138.1 were changed according to the Genome Taxonomy Database (Parks et al., 2013). Verrucomicrobia were named Verrucomicrobiota, Fusobacteria were named Fusobacteriota, Bacteroidetes were named Bacteroidota, Cyanobacteria were named Cyanobacteriota, Actinobacteria were named Actinomycetota, Comamonadaceae were named Burkholderiaceae. Firmicutes were named Bacillota, Chloroflexi were named Chloroflexota, Rhodobacteriaceae were named Rhodobacteraceae, Dechloromonas were named Azonexus, and Tychonema were named Microcoleaceae.

The Sanger sequences from the ITS2 region of the snail specimen were quality‐checked using Chromas software, merged using Bioedit (version 7.7.1.0), and submitted to NCBI GenBank under the accession numbers OR288013–OR288075 (Supporting Information Figure 1).

Sequences from this study and selected reference sequences from Kivistik et al. (2022a) were imported to the ARB Program suite (Ludwig, 2004) to calculate a maximum likelihood phylogenetic tree (PhyML). The topology of the tree was tested separately by neighbor‐joining and parsimony analysis (DNAPARS) with a bootstrapping algorithm (seqboot; 1000 bootstraps). The original sequence definitions in the GenBank database were replaced with a consistent nomenclature including accession number, species, and geographic origin.

### 2.6 Statistical analysis

The PAST software package version 3.22 (Hammer et al., 2001) was used for statistical analysis. Samples for beta diversity analysis were normalized using the Centered log‐ratio (CLR) transformation. CLR values are scale‐invariant such that the same ratio is obtained regardless of differences in read counts (Gloor et al., 2017). For Chao1 estimations, Explicet (Robertson et al., 2013) was used, which performs a rarefaction‐based analysis through bootstrapping. A Kruskal–Wallis test and a post hoc Dunn's pairwise test were used to calculate significant differences between the numbers of ASVs in the samples. Differences in the bacterial community composition were tested by permutational multivariate analysis of variance (PERMANOVA) combined with a pairwise PERMANOVA test between all pairs of groups as a post hoc test corrected by the sequential Bonferroni method. The similarity between bacterial communities was visualized by principal coordinates analysis (PCA, based on Euclidean distance) in combination with CLR‐transformed data at an Aitchison distance. The relative overlap of the ASV in the specific substrates and countries was calculated using a multiple list comparator (https://molbiotools.com/) and visualized as Venn diagrams. To test for correlations between community similarity and geographic distances between samples, the data were split by substrate. The Dice/Sørenson index was calculated for the bacterial community distance in PAST, and the geographic distance between sampling points was estimated using the spherical law of cosines formula, which accounts for the spherical nature of the Earth. The distance‐decay rate was assessed by slope of linear fitting (lm() in R) between Dice/Sørenson index and geographical distance, with statistically significant difference between slopes as tested by analysis of covariance (lstrends() in R). In addition, we tested the correlation between Dice/Sørenson index and geographical distances matrices using a Mantel test (mantel.rtest() in R).

## 3 RESULTS

### 3.1 Bacterial community composition

**Figure 2:**
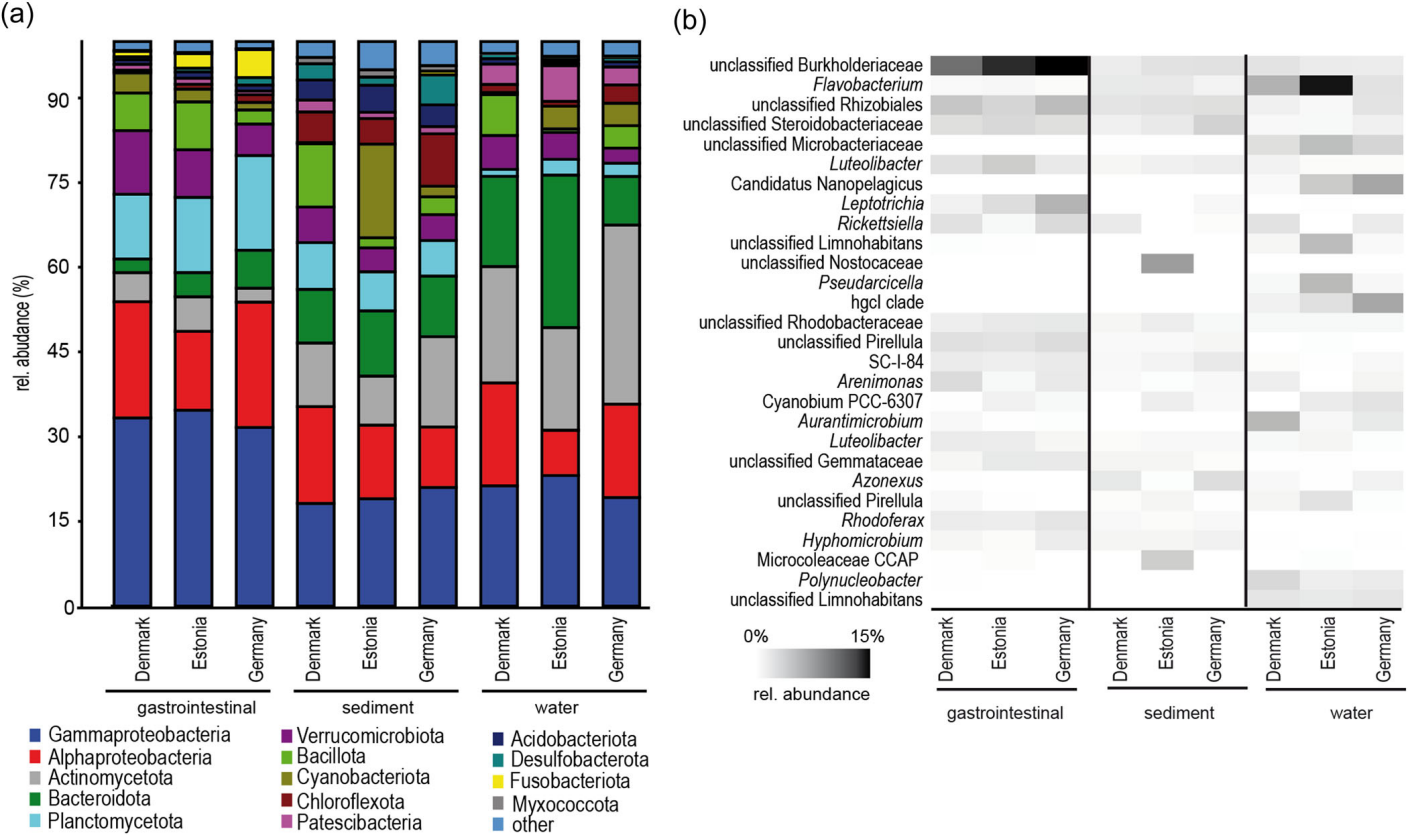
Relative abundance of (a) bacterial phyla/classes and (b) most abundant (>0.5%) amplicon sequence variants.

On a broad phylogenetic level (Figure 2a), abundant taxa were present in snail gastrointestinal samples, sediment, and water. However, abundances differed between the substrates and between the different countries. In the gastrointestinal tract, Gammaproteobacteria (31%–34%) and Alphaproteobacteria (14%–22%) were the most abundant classes, followed by Planctomycetota (11%–17%) and Verrucomicrobiota (6%–11%). Fusobacteriota had the highest relative abundance in samples from Germany (5%) and the lowest in samples from Denmark (1%). In the sediment samples Gammaproteobacteria (18%–21%), Alphaproteobacteria (11%–17%), Bacteroidota (10%–12%), and Cyanobacteriota (0%–17%) were also the most abundant. These abundant phyla were irregularly distributed between countries, including Actinomycetota (9%–16%) and Bacillota (2%–11%). Cyanobacteriota had a high abundance in the samples from Estonia (17%) and were missing in Denmark (0%). Except for the Gammaproteobacteria (19%–21%), the abundant phyla/classes in the water samples were also irregularly distributed, including Actinomycetota (18%–31%), Bacteroidota (9%–27%), and Alphaproteobacteria (8%–17%). Bacteroidota had a high abundance in Estonia (27%) but low in Germany (9%). In addition, at the finest taxonomic level (ASV), a clear difference in the abundance of ASVs between the three substrates and countries was obvious (Figure 2b). Unclassified Burkholderiaceae were most abundant in snails, with the lowest abundances being in Denmark. The genus *Flavobacterium* was almost absent in snails but highly abundant in water samples, especially in Estonia. Unclassified Microbacteriaceae and *Candidatus* Nanopelagicus were only found in the water samples, whereas the highest abundances of *Candidatus* Nanopelagicus were found in Germany and unclassified Microbacteriaceae in Estonia. Unclassified ASVs of the genera *Pirellula*, *Luteolibacter*, *Cutibacterium*, and *Candidatus* Saccharimonas were found in 90% of the *A. balthica* samples, of which *Pirellula* and *Luteolibacter* were abundant (Figure 2b).

**Figure 3:**
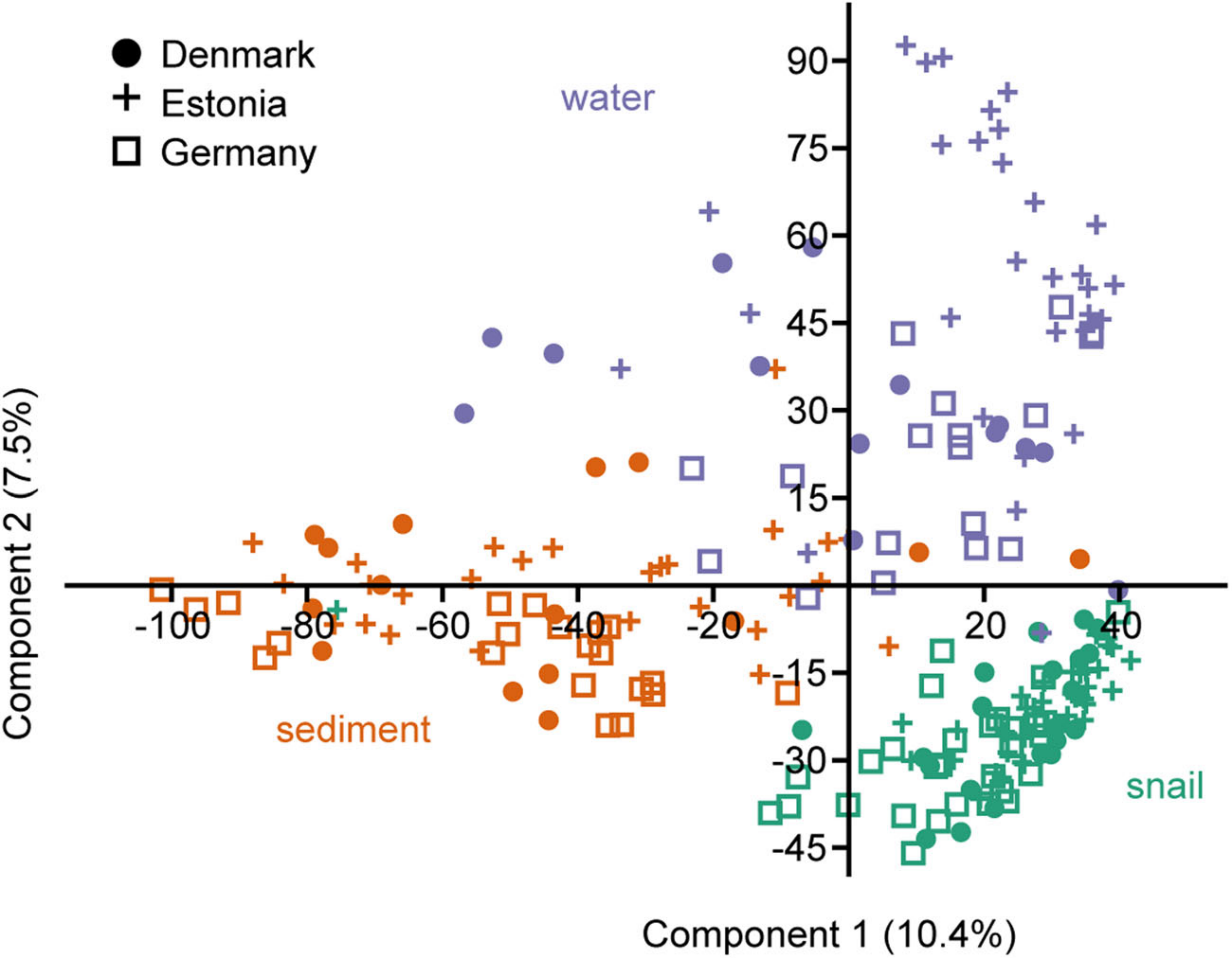
Principle coordinates analysis of the bacterial community composition in sediment (orange), water (violet), and snail gastrointestinal tract bacterial community (green).

**Figure 4:**
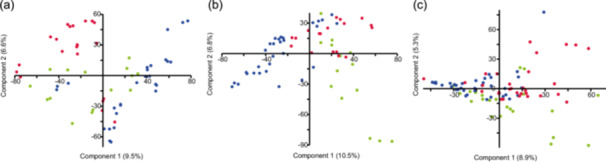
Principle coordinates analysis of the bacterial community composition in Denmark (green), Estonia (blue), and Germany (red) in (a) sediment, (b) water, and (c) gastrointestinal tract bacterial community.

Accordingly, the PCA of all samples showed a separation of the bacterial community depending on the substrate, that is, sediment, water, and gastrointestinal tract (Figure 3). PERMANOVA analysis supported a significant difference between water versus sediment versus gastrointestinal tract‐associated bacterial communities (Supporting Information Table 1) and pairwise PERMANOVA between all samples indicated *p* < 0.01 for all comparisons. Therefore, the three substrates were analyzed separately in the following analysis. Comparison of the separated gastrointestinal, sediment, and water bacterial community composition between countries showed a significant difference between the country‐specific bacterial communities (Figure 4 and Supporting Information Tables 2 and 4–6) also in the pairwise comparison. The bacterial communities within a substrate varied between the sampling stations of a country (Supporting Information Table 3). However, the more conservative pairwise comparison did not reveal significant differences between the sites of a country (Supporting Information Tables 7–15), suggesting a greater difference in the bacterial community composition between the countries compared with that within countries.

To explore biogeographic differences in microbial communities along the longitudinal gradient, community similarity was plotted as a function of geographic distance between sampling points (Supporting Information Figure 2). The geographic distance was, on average, 625 km, between the farthest sampling points was 1245 km and the smallest distance was 2 km. The slopes between the host‐associated and free‐living bacterial communities differed significantly and gastrointestinal tract bacterial community distance had the lowest *r* value in the Mantel test (Supporting Information Tables 16 and 17). The turnover rate (i.e., the rate at which species composition changes with geographic distance (Soininen et al., 2007)) was the highest for water samples (0.051 per 1000 km) and sediment (0.027 per 1000 km) and smallest for snail gastrointestinal microbiome samples (0.013 per 1000 km). However, the distance was not continuous, and samples between 300 and 900 km were missing (Supporting Information Figure 2).

### 3.2 Number of bacteria within one substrate/country

**Figure 5:**
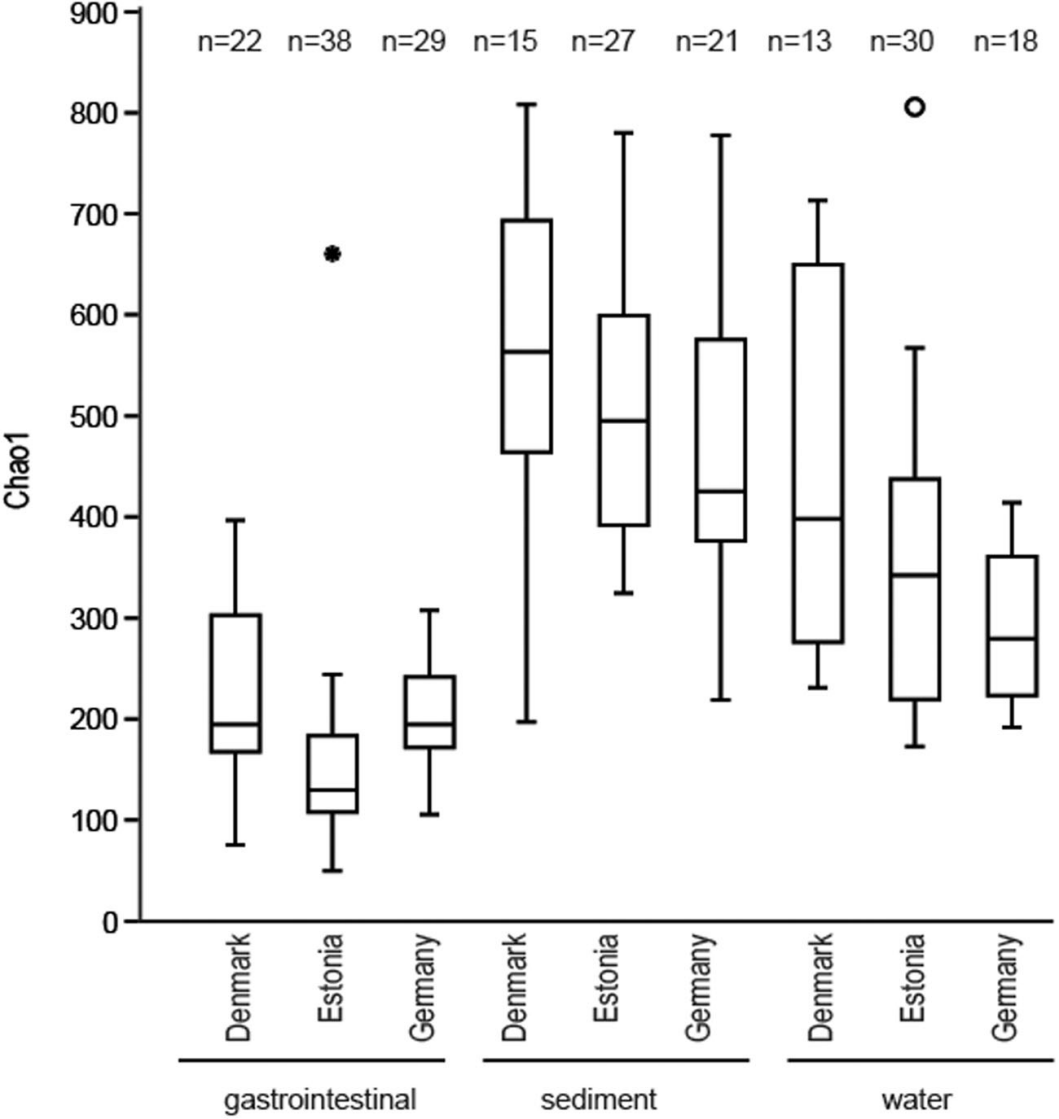
Boxplot of Chao1 diversity. Samples are grouped according to substrate, and country of origin. The box of the plots indicates the 25%–75% quartiles; the median is shown by a horizontal line inside the box. The minimal and maximal values are shown with short vertical lines. Values outside the inner fences are shown as circles, and values further than 3× the box height from the box (“outer fences”) are shown as stars.

The average Chao1 diversity of the gastrointestinal microbiome was significantly lower than the average sediment and the average water Chao1 diversity except for water from Germany (Supporting Information Table 17 and Figure 5). The water bacterial Chao1 richness from Estonia and Germany differed significantly from that of sediment. To differentiate substrate/country‐specific ASVs, Venn diagrams were prepared. There was a significant difference between the relative abundance of ASVs in Denmark, Estonia, and Germany within the water, snail gastrointestinal tract, and sediment. Also, a significant distance existed between the gastrointestinal tract bacterial community, water, and sediment in Denmark, Estonia, and Germany.

**Figure 6:**
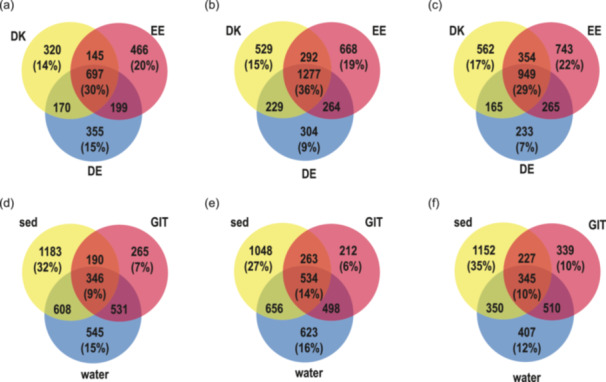
Venn diagrams of bacterial community's proportions in (a) snail gastrointestinal tract, (b) sediment, (c) water, (d) Denmark (DK), (e) Estonia (EE), and (f) Germany (DE). GIT, gastrointestinal tract; sed, sediment.

The snail gastrointestinal, water and sediment bacterial communities shared 30%, 29%, and 36% of ASVs between the three countries (Figure 6a–c). Within a country, the snail gastrointestinal tract bacterial community composition always had the fewest specific ASVs (Denmark 7%, Estonia 6%, and Germany 10%), whereas sediment always had the highest percentage of specific ASVs (Denmark 32%, Estonia 27%, and Germany 35%) (Figure 6d–f). In addition, the overlap of ASV between the substrates in one country was relatively low (Denmark 9%, Estonia 14%, and Germany 10%). The highest number of ASVs overlapping with the gastrointestinal bacterial community was the water bacterial community (Denmark 15%, Estonia 13%, and Germany 15%). In addition to the difference between country and substrate, we observed differences in the bacterial communities depending on whether the sample was taken in flowing water (river) or standing water (lake, pond) in all samples (PERMANOVA, *F* = 3.23, *p* < 0.01). PCA and statistical tests between standing and flowing water of the gastrointestinal tract (PERMANOVA, *F* = 2.1, *p* < 0.01), sediment (PERMANOVA, *F* = 2.2, *p* < 0.01), and water bacterial community (PERMANOVA, *F* = 4.1, *p* < 0.01) supported this result (Supporting Information Figure 3). However, within a water type (flowing or standing water) the country and substrate bacterial community composition were significantly different (standing water, two‐way PERMANOVA *country F* = 3.18 *p* < 0.01; *substrate F* = 5.17 *p* < 0.01; flowing water, two‐way PERMANOVA *country F* = 4.13 *p* < 0.01; *substrate F* = 15.2 *p* < 0.01). Because the two environments were relatively evenly distributed in the countries (Table 1), the effect of flowing and standing water did not influence the country‐dependent analysis.

### 3.3 Host identification through phylogenetic marker genes

To support the morphological identification and exclude cryptic species in the different geographic regions of *A. balthica*, we randomly analyzed the ITS2 biomarker regions of 62 snail samples. Our analysis confirmed that the snails sampled in Germany, Denmark, and Estonia belong to *A. balthica* with highly identical ITS sequences (99% identity, Supporting Information Figure 1) and a comparable genetic host basis can be expected.

## 4 DISCUSSION

In this study, we investigated field‐collected gastrointestinal bacterial communities of *A. balthica* specimens and their surrounding water and sediment bacterial communities from Estonia, Denmark, and northern Germany using culture‐independent molecular analysis. Estonia belongs to the boreal region, while the northeastern part of Germany and Denmark belong to the Atlantic/Continental geographical region (Habitats Directive 92/43/EEC). These differences are also reflected in the increase in the geographic distance between the samples. The bacterial community structure of the sediment and water showed stronger differences with increasing distance compared with the gastrointestinal bacterial community. This supports the hypothesis that host‐associated bacterial communities have a lower distance‐decay relationship and act as habitat islands. The distance‐decay relationship of the bacterial communities quantifies changes in community similarity with increased geographic distance (Soininen et al., 2007). Generally, geographically closer bacterial communities are expected to be more similar, as indicated in lower turnover rates (Soininen et al., 2007) if conditions between the samples are comparable. The low turnover rates of the gastrointestinal tract indicated that the similarity of the gastrointestinal tract microbiome is more constant at larger distances compared with water or sediment bacterial communities. Hence, the gastrointestinal tract provides unique conditions for selecting specific bacterial communities rather than environmental factors (Suzzi et al., 2023; Taylor et al., 2005). However, we sampled only comparable environments (lakes, rivers) on a relatively small geographic scale (max 1245 km). Environmental gradients and different biogeographic regions (e.g., salinity or temperature) may result in higher distance‐decay relationships but measuring effects within comparable biogeographic regions allows one to exclude strong environmental effects (e.g., desert vs. arctic). In addition, the taxonomic resolution based on 450 bp of the 16S rRNA gene may not be sufficient to distinguish fine ASV differences.

Between the three countries, approximately 1/3 ASV were shared within one substrate, whereas the sediment bacterial community had the highest overlap between countries (Figure 6a–c). The water and gastrointestinal bacterial communies had the highest overlap, suggesting that water is an important source for the gastrointestinal bacterial community composition (Figure 6d,e). Water as an important source for the snails' gastrointestinal trace was rather unexpected because snails graze on sediment biofilm and a higher share of sediment bacteria would have been expected. The gastrointestinal tract had the lowest percentage of specific ASVs per country, indicating that many of the gastrointestinal bacteria also occur in the environment and represent facultative symbionts. Overall, the results support that the gastrointestinal tract bacterial community is based on a combination of host characteristics and environmental bacteria (Loo et al., 2019). Since vertical transmission of gastrointestinal bacteria is not described for *A. balthica*, an important mechanism for colonization of the gastrointestinal tract is the “priority effect” (Walter & Ley, 2011). Bacterial populations establish during the early development of the symbiosis, and outcompete later arrivals (Burr et al., 2022). If these bacteria can colonize the gastrointestinal tract permanently, they create a local gastrointestinal microbiome that is specific to the gastrointestinal tract but shows a clear overlap with sediment and water bacteria. The concept of a selection from the local environment is supported by comparison with a previous study in Estonia, where high abundances of *Mycoplasma*, *Luteolibacter*, and *Aeromonas* have been found in *A. balthica* gastrointestinal communities (Kivistik et al., 2022a). These bacterial genera were also found to be highly abundant in our samples from Estonia (*Mycoplasma* [2.0%], *Luteolibacter* [3.1%], and *Aeromonas* [3.5%]) despite being sampled in different years, rivers, and lakes. However, these ASVs were only lowly abundant in the samples from Denmark (*Mycoplasma* [0.1%], *Luteolibacter* [1.9%], and *Aeromonas* [0.1%]) and Germany (*Mycoplasma* [0.1%], *Luteolibacter* [1.3%], and *Aeromonas* [0.0%]) suggesting that they were not available during initial colonization. A strong influence of environmental factors and low turnover rates were also observed in previous studies of gastrointestinal bacterial communities in estuarine fishes (Suzzi et al., 2023).

As expected, based on the results reported in previous invertebrate gut microbiome analysis (Sullam et al., 2015; Suzzi et al., 2023) the number of ASV in the gastrointestinal tract did not differ significantly between samples from different biogeographic regions (Figure 5), but were lower compared with water or sediment. In all gastrointestinal samples, Gammaproteobacteria (23.3%–46.3%), Alphaproteobacteria (13.0%–22.8%), and Planctomycetes (3.0%–16.6%) were dominant. The high abundance of Gamma‐ and Alphaproteobacteria in *A. balthica* is consistent with previous studies that have shown the dominance of these classes within the host associate microbiota of *Radix auricularia* (Hu et al., 2018) and other snails, including *Achatina fulica* (Pawar et al., 2012), *Helisoma duryi* (Van Horn et al., 2012), *Helix pomatia* (Nicolai et al., 2015), *Biomphalaria pfeifferi*, *Bulinus africanus*, and *H. duryi* (Van Horn et al., 2012).

In contrast to the gastrointestinal tract, the sediment communities were characterized by higher species richness, the highest percentage of specific ASVs in each country (Figure 6d–f), and high overlap of ASVs between countries (Figure 6b). This can be due to the complex and heterogeneous gradients of substrate, pH, and redox potential gradients of sediments, forming higher habitat heterogeneity in sediments that select for specific bacterial communities (Jørgensen & Boetius, 2007; Torsvik et al., 2002).

Our study confirmed that biogeographical patterns in host‐associated communities differ from those in water and sediment bacterial communities and support the idea that they act like habitat islands. The gastrointestinal bacterial community was determined by a combination of host‐specific and environmental bacteria indicating that environmental changes, especially pulse events from climate change or industrial pollution, will have a strong impact on the host‐associated microbiome (Iglesias, 2020).

## AUTHOR CONTRIBUTIONS

**Daniel P. R. Herlemann**: Conceptualization; formal analysis; funding acquisition; resources; writing—original draft; writing—review and editing; investigation; and data curation. **Helen Tammert**: Writing—original draft; writing—review and editing; validation; resources; investigation; and data curation. **Carmen Kivistik**: Resources and investigation. **Kairi Käiro**: Resources; investigation; and data curation. **Veljo Kisand**: Software; formal analysis; writing—original draft; writing—review and editing; and visualization.

## CONFLICT OF INTEREST STATEMENT

None declared.

## ETHICS STATEMENT

None required.

## Supporting information

Supporting information.

## Data Availability

The raw reads from Illumina sequencing were deposited at the NCBI in BioProject PRJNA1028832 under the accession numbers SAMN38726042–SAMN38726255. The Sanger sequences from the ITS2 region of the snail specimen were submitted to NCBI GenBank under the accession numbers OR288013–OR288075.
