# Supplementary material for: Distinct biogeographical patterns in snail gastrointestinal tract bacterial communities compared with sediment and water
Source: Microbiologyopen. 2024 Jun 2;13(3):e13. doi: 10.1002/mbo3.1413 (PMC11144953; doi:10.1002/mbo3.1413)
Supplement: Supplementary file 1 — Supporting information. [file MBO3-13-e13-s001.pdf]

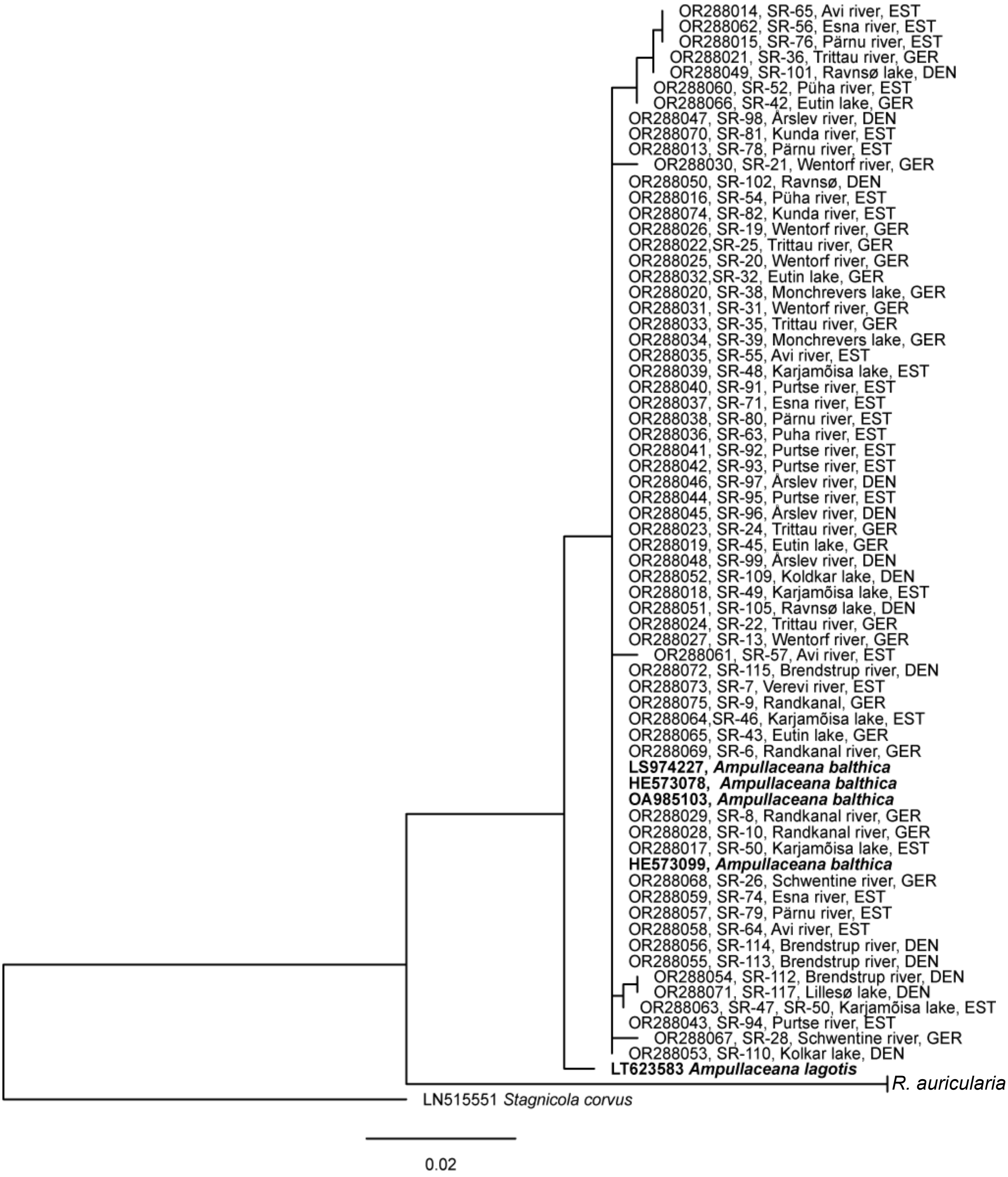


**Supplementary Figure 1.** Nuclear marker internal transcribed spacer 2 (ITS2) sequence maximum likelihood tree based on 295 sequence columns with *Stagnicola corvus and Radix auricularia* as outgroup. Sequences with NCBI acc. number LS974227, HE573078, OA985103 and HE573099 from *Ampullaceana balthica* and a sequence of *Ampullaceana lagotis* (NCBI acc. Number LT623583) of previous studies were used as reference (marked in bold). Sequences with the NCBI acc number OR288013 -OR288075 are from this study. (DEN= Denmark; EST=Estonia; GER= Germany)

(A) Sediment

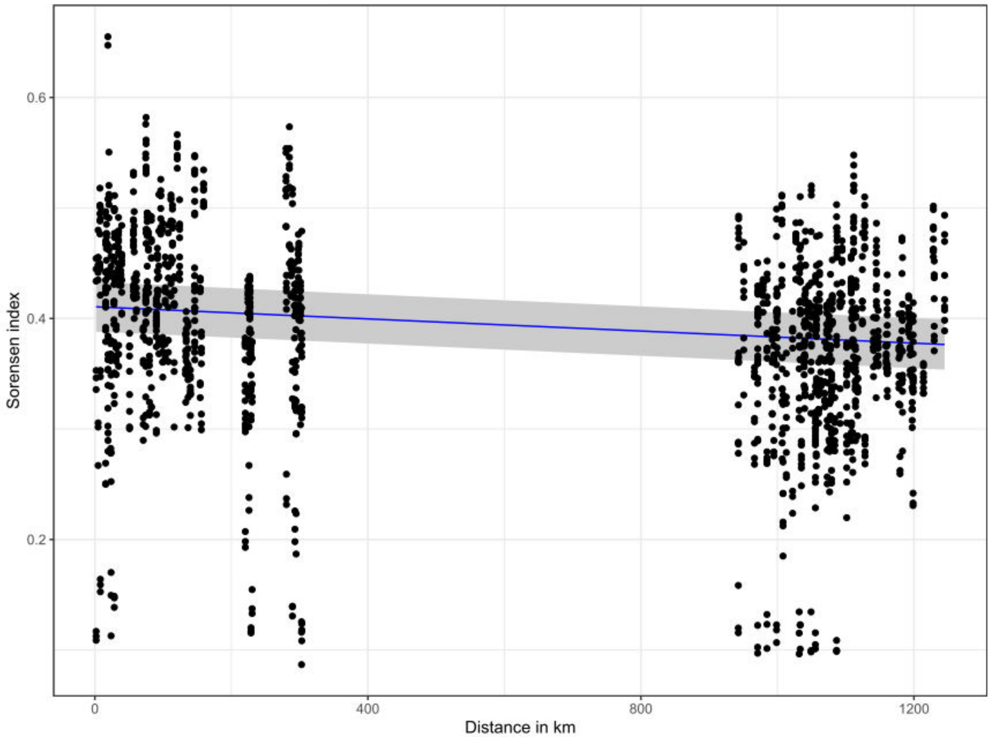


(B) Water

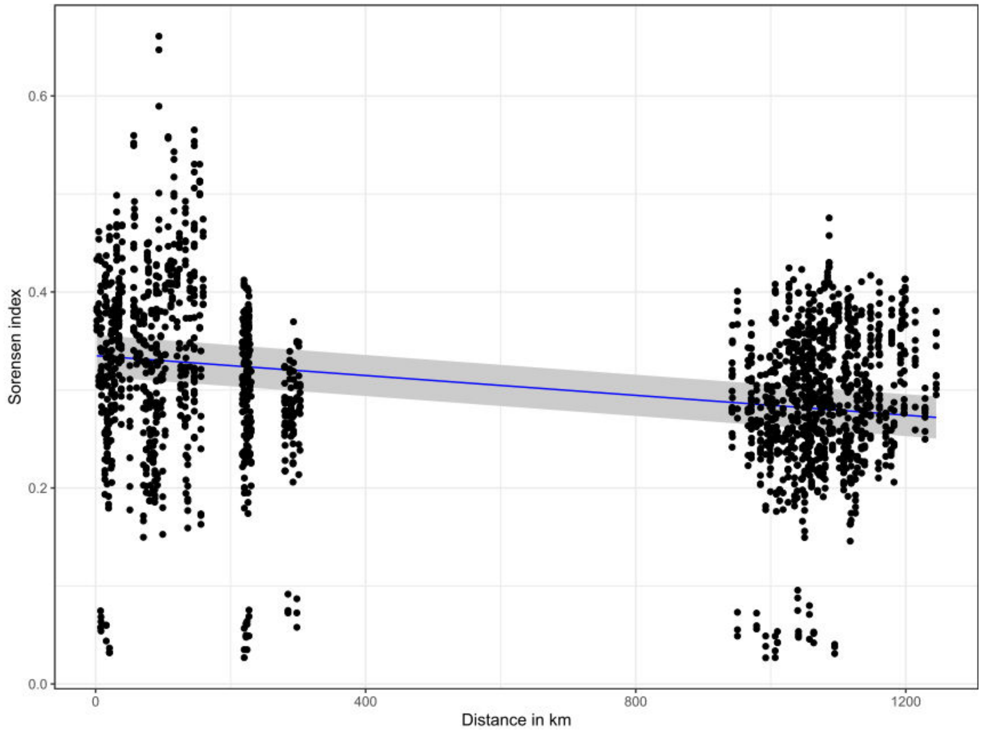

(C) Gastrointestinal tract

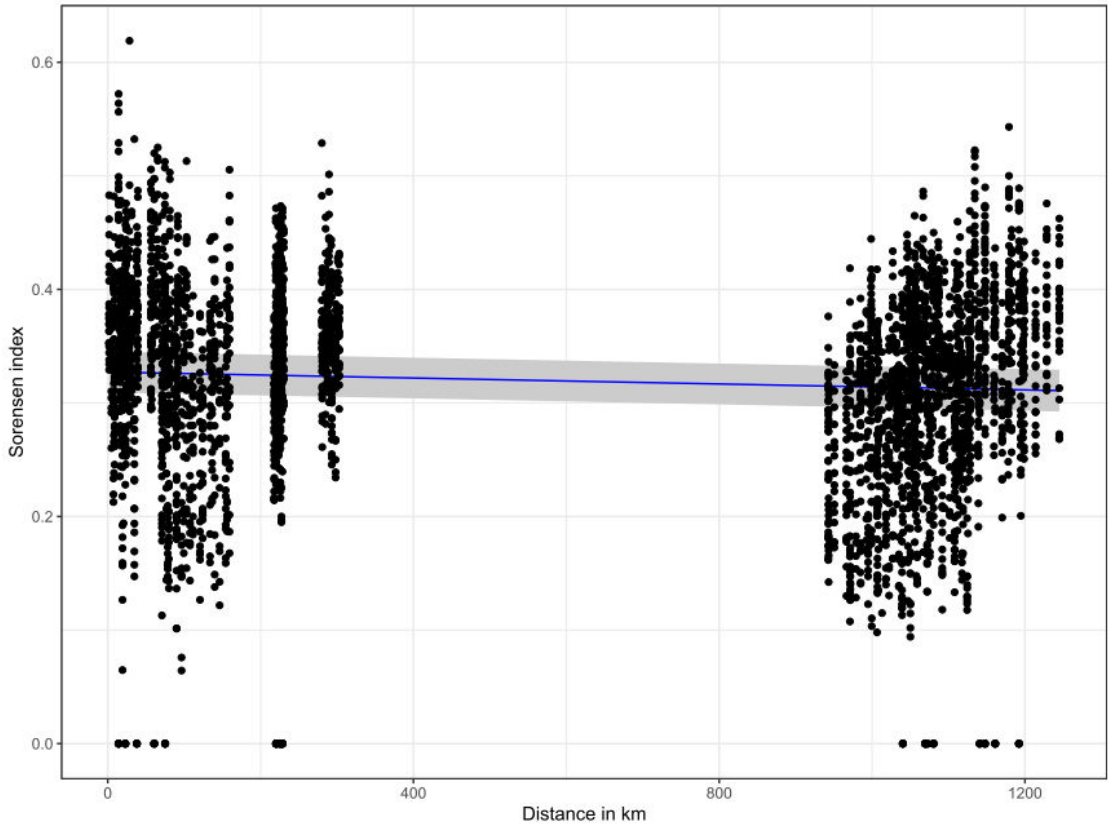


**Supplementary Figure 2.** Distance decay relationship between (A) sediment, (B) water and (C) snail gastrointestinal tract samples. The phylogenetic distance is based on the Dice/Sørenson index and the geographic distance between sampling points was estimated using the spherical law of cosines formula, which accounts for the spherical nature of the Earth. The grey areas gives the 95% confidence.

(A) Sediment samples

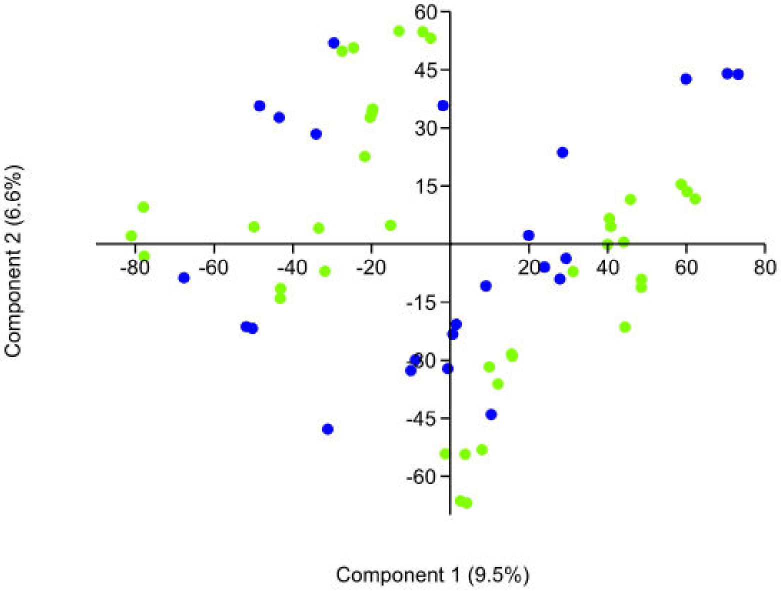


(B) Water samples

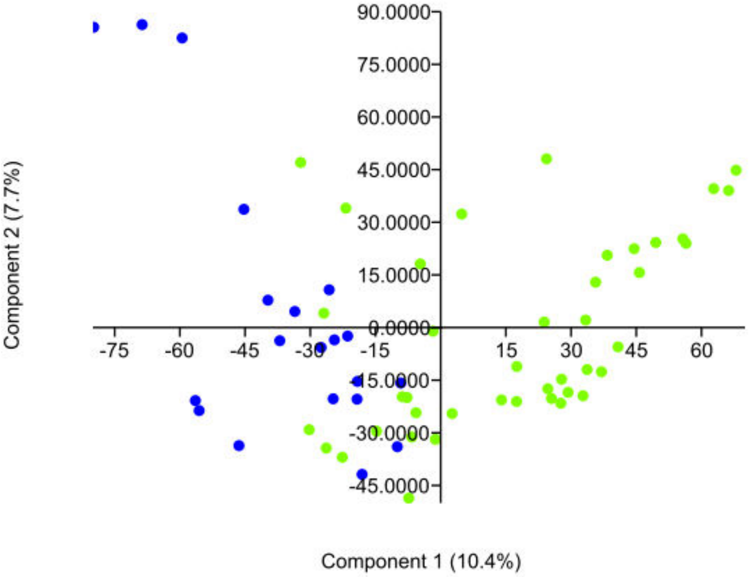

(C) Gastrointestinal tract

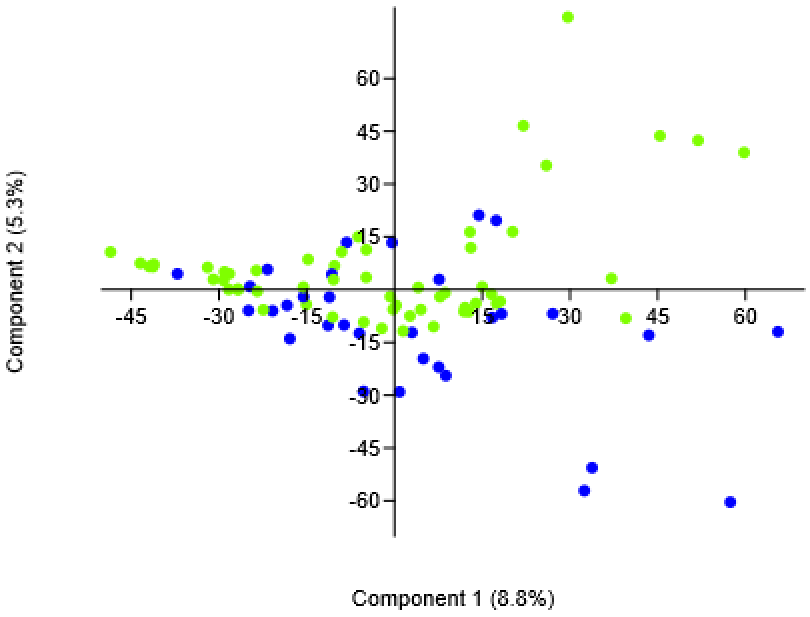


**Supplementary Figure 3.** Principle component analysis of the bacterial community composition of the (A) sediment, (B) water and  (c) gastrointestinal tract colored by running water (green) and standing water (blue) bacterial communities.

.

A. Sediment samples rarefraction

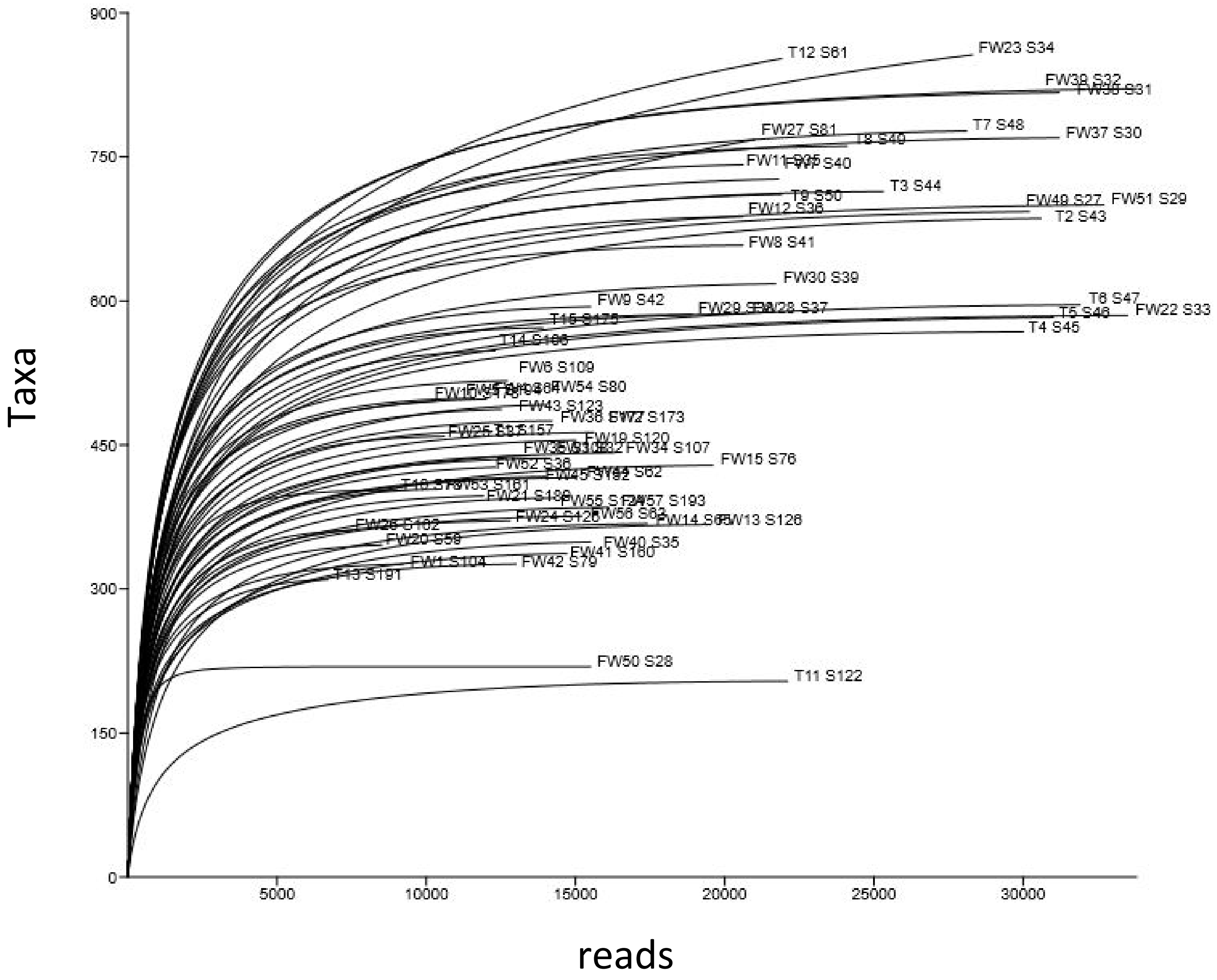

B. Water samples rarefraction

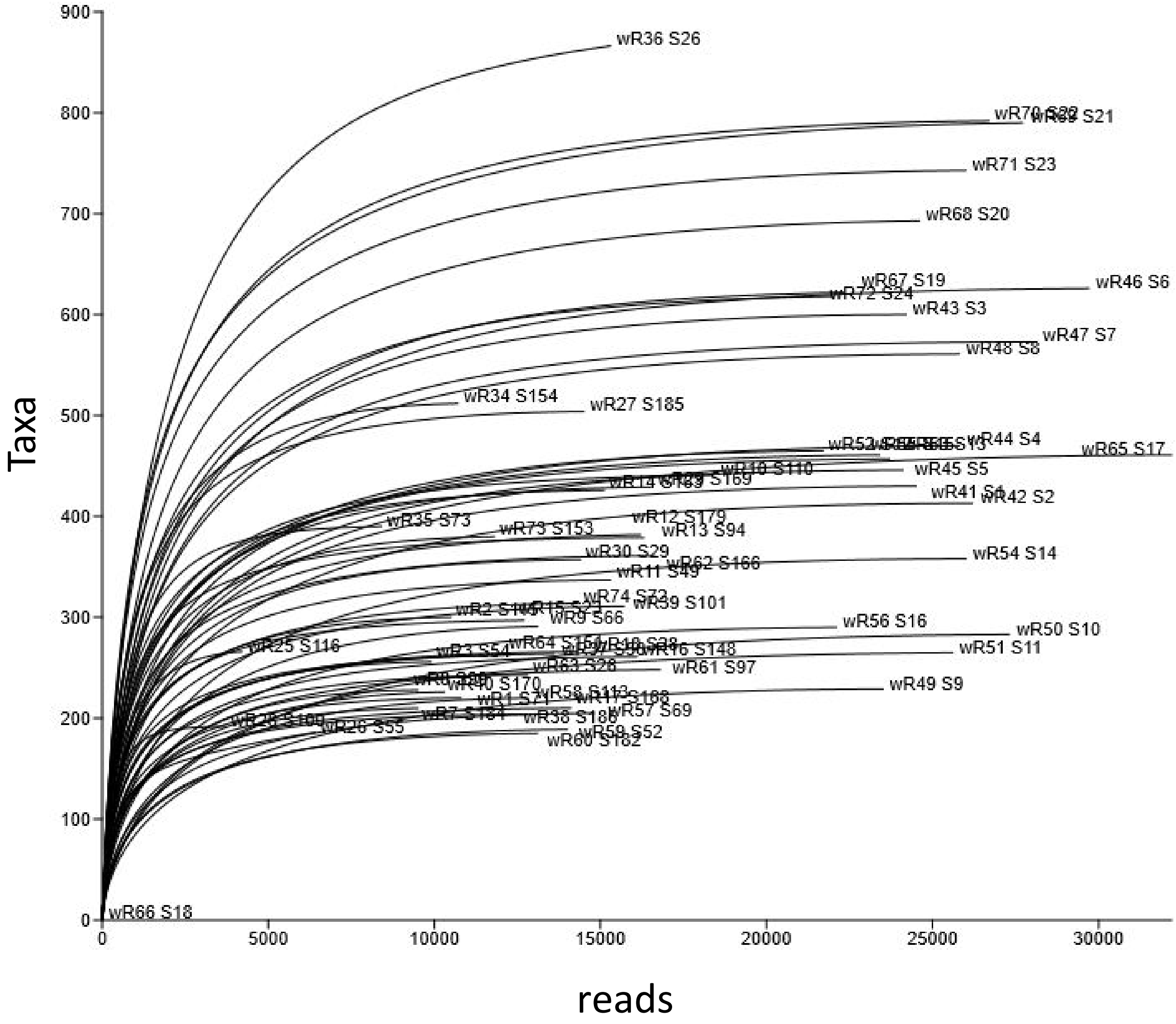

C. Gastrointestinal tract samples rarefraction

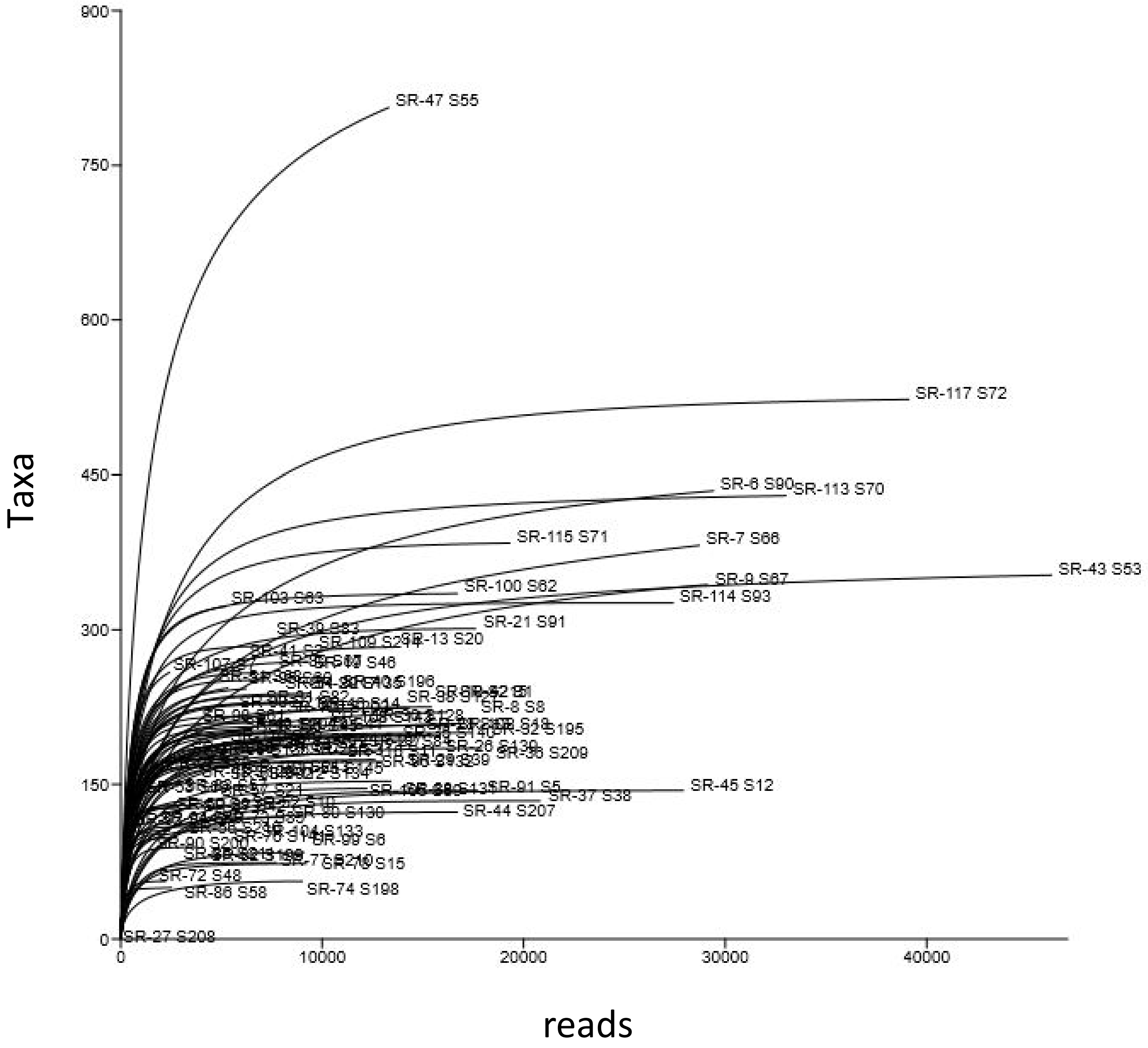


**Supplementary Figure 4.** Sample rarefaction curves (A) sediment, (B) water and (c) gastrointestinal tract bacterial communities.

Supplementary Table 1. Results of a two-way PERMANOVA of all samples testing differences in the bacterial community in the substrates, country, and its interaction.

| | df | F-value | p-value |
|---|---|---|---|
| Substrate | 2 | 16.9 | p<0.01 |
| Country | 2 | 5.6 | p<0.01 |
| Interaction | 4 | 2.7 | p<0.01 |

Supplementary Table 2. Results of one-way PERMANOVA testing differences in the bacterial community in the substrates and country (the tested group is in brackets, GIT=gastrointestinal tract).

| | df | F-value | p-value |
|---|---|---|---|
| Substrate (all samples) | 2 | 15.7 | p<0.01 |
| Country (water) | 2 | 3.6 | p<0.01 |
| Country (GIT) | 2 | 3.2 | p<0.01 |
| Country (sediment) | 2 | 3.5 | p<0.01 |
| Substrate (Denmark) | 2 | 4.6 | p<0.01 |
| Substrate (Estonia) | 2 | 11.1 | p<0.01 |
| Substrate (Germany) | 2 | 6.8 | p<0.01 |

Supplementary Table 3. Results of one-way PERMANOVA testing differences at bacterial community in the different sampling sites (GIT=gastrointestinal tract).

| | df | F-value | p-value |
|---|---|---|---|
| Water Denmark (sites) | 3 | 2.2 | p<0.01 |
| Water Estonia (sites) | 6 | 2.3 | p<0.01 |
| Water Germany (sites) | 4 | 3.1 | p<0.01 |
| Sediment Denmark (sites) | 3 | 2.5 | p<0.01 |
| Sediment Estonia (sites) | 6 | 3.0 | p<0.01 |
| Sediment Germany (sites) | 4 | 2.1 | p<0.01 |
| GIT Denmark (sites) | 3 | 1.6 | p<0.01 |
| GIT Estonia (sites) | 5 | 1.5 | p<0.01 |
| GIT Germany (sites) | 4 | 2.7 | p<0.01 |

Supplementary Table 4. Results of a pairwise comparison one-way PERMANOVA testing differences in countries of the sediment bacterial community (p-values, Bonferroni corrected).

| | Estonia | Germany |
|---|---|---|
| Denmark | <0.01 | <0.01 |
| Estonia | | <0.01 |

Supplementary Table 5. Results of a pairwise comparison one-way PERMANOVA testing differences in countries of the gastrointestinal bacterial community (p-values, Bonferroni corrected).

| | Estonia | Germany |
|---|---|---|
| Denmark | <0.01 | <0.01 |
| Estonia | | <0.01 |

Supplementary Table 6. Results of a pairwise comparison one-way PERMANOVA testing differences in countries of the water bacterial community (p-values, Bonferroni corrected).

| | Estonia | Germany |
|---|---|---|
| Denmark | <0.01 | <0.01 |
| Estonia | | <0.01 |

Supplementary Table 7. Results of a pairwise comparison one-way PERMANOVA testing differences in sites of sediment bacterial community in Denmark (p-values, Bonferroni corrected).

| | Koldkar | Ravnsø | Brendstrup | Lillesø |
|---|---|---|---|---|
| Årslev | 0.99 | 0.98 | 0.98 | 1 |
| Koldkar | | 0.98 | 1 | 1 |
| Ravnsø | 0.98 | | 0.98 | 1 |
| Brendstrup | 1 | 0.98 | | 1 |

Supplementary Table 8. Results of a pairwise comparison one-way PERMANOVA testing differences in sites of sediment bacterial community in Estonia (p-values, Bonferroni corrected).

| | Pühajõgi | Pärnu | Võrtsjärv | Kunda | Esna | Purtse | Ahja |
|---|---|---|---|---|---|---|---|
| Avijõgi | 1 | 1 | 1 | 1 | 1 | 1 | 1 |
| Pühajõgi | | 1 | 1 | 1 | 1 | 1 | 1 |
| Pärnu | 1 | | 1 | 1 | 1 | 1 | 1 |
| Võrtsjärv | 1 | 1 | | 1 | 1 | 1 | 1 |
| Kunda | 1 | 1 | 1 | | 1 | 1 | 1 |
| Esna | 1 | 1 | 1 | 1 | | 1 | 1 |
| Purtse | 1 | 1 | 1 | 1 | 1 | | 1 |

Supplementary Table 9. Results of a pairwise comparison one-way PERMANOVA testing differences in sites of sediment bacterial community in Germany (p-values, Bonferroni corrected).

| | Ratzeburg | Lütau | Wentorf | Randkanal | Trittau |
|---|---|---|---|---|---|
| Schwentine | 1 | 1 | 1 | 1 | 1 |
| Ratzeburg | | 1 | 1 | 1 | 1 |
| Lütau | 1 | | 1 | 1 | 1 |
| Wentorf | 1 | 1 | | 1 | 1 |
| Randkanal | 1 | 1 | 1 | | 1 |

Supplementary Table 10. Results of a pairwise comparison one-way PERMANOVA testing differences in sites of the gastrointestinal bacterial community in Denmark (p-values, Bonferroni corrected).

| | Ravnsø | Koldkar | Brendstrup | Lillesø |
|---|---|---|---|---|
| Årslev | 0.84 | 1 | 0.25 | 0.92 |
| Ravnsø | | 0.08 | 0.09 | 0.48 |
| Koldkar | 0.08 | | 0.06 | 0.97 |
| Brendstrup | 0.09 | 0.06 | | 0.52 |

Supplementary Table 11. Results of a pairwise comparison one-way PERMANOVA testing differences in sites of the gastrointestinal bacterial community in Estonia (p-values, Bonferroni corrected).

| | Võrtsjärv | Kunda | Pärnu | Esna | Karjamõisa | Pühajõgi | Avijõgi |
|---|---|---|---|---|---|---|---|
| Purtse | 1 | 0.73 | 0.23 | 1 | 0.22 | 0.42 | 1 |
| Võrtsjärv | | 1 | 1 | 1 | 0.18 | 0.21 | 0.63 |
| Kunda | 1 | | 0.95 | 1 | 0.47 | 1 | 1 |
| Pärnu | 1 | 0.95 | | 1 | 0.22 | 0.27 | 0.72 |
| Esna | 1 | 1 | 1 | | 0.69 | 0.41 | 0.66 |
| Karjamõisa | 0.18 | 0.47 | 0.22 | 0.69 | | 0.24 | 0.26 |
| Pühajõgi | 0.21 | 1 | 0.27 | 0.41 | 0.24 | | 0.89 |

Supplementary Table 12. Results of a pairwise comparison one-way PERMANOVA testing differences in sites of the gastrointestinal bacterial community in Germany (p-values, Bonferroni corrected).

| | Eutin | Monchrevers | Trittau | Wentorf | Schwentine |
|---|---|---|---|---|---|
| Randkanal | 0.12 | 0.12 | 0.11 | 0.11 | 0.15 |
| Eutin | | 0.14 | 0.12 | 0.11 | 1 |
| Monchrevers | 0.14 | | 0.12 | 0.12 | 0.12 |
| Trittau | 0.12 | 0.12 | | 0.10 | 0.12 |
| Wentorf | 0.11 | 0.12 | 0.10 | | 0.09 |

Supplementary Table 13. Results of a pairwise comparison one-way PERMANOVA testing differences in sites of the water bacterial community in Denmark (p-values, Bonferroni corrected).

| | Lillesø | Årslev | Koldkar | Brendstrup |
|---|---|---|---|---|
| Ravnsø | 1 | 1 | 0.98 | 1 |
| Lillesø | | 0.99 | 1 | 0.97 |
| Årslev | 0.99 | | 0.99 | 1 |
| Koldkar | 1 | 0.99 | | 1 |

Supplementary Table 14. Results of a pairwise comparison one-way PERMANOVA testing differences in sites of the water bacterial community in Estonia (p-values, Bonferroni corrected).

| | Avijõgi | Karjamõisa | Verevi | Pärnu | Purtse | Pühajõgi | Esna |
|---|---|---|---|---|---|---|---|
| Kunda | 1 | 1 | 1 | 1 | 1 | 1 | 1 |
| Avijõgi | | 1 | 1 | 1 | 1 | 1 | 1 |
| Karjamõisa | 1 | | 1 | 1 | 1 | 1 | 1 |
| Verevi | 1 | 1 | | 1 | 1 | 1 | 1 |
| Pärnu | 1 | 1 | 1 | | 1 | 1 | 1 |
| Purtse | 1 | 1 | 1 | 1 | | 1 | 1 |
| Pühajõgi | 1 | 1 | 1 | 1 | 1 | | 1 |

Supplementary Table 15. Results of a pairwise comparison one-way PERMANOVA testing differences in sites of the water bacterial community in Germany (p-values, Bonferroni corrected).

| | Trittau | Schwentine | Monchrevers | Wentorf | Eutin |
|---|---|---|---|---|---|
| Randkanal | 1 | 1 | 1 | 1 | 1 |
| Trittau | | 1 | 1 | 1 | 1 |
| Schwentine | 1 | | 1 | 1 | 1 |
| Monchrevers | 1 | 1 | | 1 | 1 |
| Wentorf | 1 | 1 | 1 | | 1 |

Supplementary Table 16. Results of a Mantel Test investigating distance vs substrate (GIT =gastrointestinal tract).

| | r-value | p-value |
|---|---|---|
| Water-distance | 0.15 | <0.01 |
| Sediment-distance | 0.20 | <0.01 |
| GIT-distance | 0.09 | <0.01 |

Supplementary Table 17. Comparison of slopes of the distance decay relationship in Supplementary Figure 2 (GIT=gastrointestinal tract).

| | Estimate | SE | z-ratio | p-value |
|---|---|---|---|---|
| Sediment-GIT | -1.40e-05 | 5.32e-06 | -2.63 | 0.02 |
| Sediment-water | 1.88e-06 | 6.04e-06 | 0.31 | 0.95 |
| GIT-water | 1.59e-05 | 4.96e-06 | 3.20 | <0.01 |

Supplementary Table 18. Results of a one-way PERMANOVA comparing the bacterial community composition in standing *vs*. flowing water (GIT=gastrointestinal tract).

| | F-value | p-value |
|---|---|---|
| all samples | 3.2 | <0.01 |
| GIT | 2.1 | <0.01 |
| water | 4.1 | <0.01 |
| sediment | 2.2 | <0.01 |
| all samples | 3.2 | <0.01 |

Supplementary Table 19. Results of the Tukey test based on the Chao1 bacterial diversity shown in Figure 3. Tukey's Q is below the diagonal, p is above the diagonal (GIT=gastrointestinal tract, Sed= sediment)

| | | GIT | | | Sediment | | | water | | |
|---|---|---|---|---|---|---|---|---|---|---|
| | | DK | EE | DE | DK | EE | DE | DK | EE | DE |
| GIT | DK | | 0.55 | 0.99 | <0.01 | <0.01 | <0.01 | <0.01 | <0.01 | 0.6573 |
| | EE | 2.81 | | 0.79 | <0.01 | <0.01 | <0.01 | <0.01 | <0.01 | <0.01 |
| | DE | 0.68 | 2.27 | | <0.01 | <0.01 | <0.01 | <0.01 | <0.01 | 0.2895 |
| Sed | DK | 11.75 | 15.37 | 12.97 | | 0.94 | 0.43 | 0.40 | <0.01 | <0.01 |
| | EE | 11.71 | 16.36 | 13.3 | 1.76 | | 0.97 | 0.93 | <0.01 | <0.01 |
| | DE | 9.49 | 13.42 | 10.78 | 3.06 | 1.60 | | 1 | <0.01 | <0.01 |
| water | DK | 7.84 | 10.89 | 8.801 | 3.13 | 1.832 | 0.42 | | 0.20 | <0.01 |
| | EE | 5.44 | 9.34 | 6.60 | 7.60 | 6.911 | 4.81 | 3.67 | | 0.77 |
| | DE | 2.59 | 5.52 | 3.39 | 8.88 | 8.34 | 6.441 | 5.27 | 2.35 | |
